# Teaching interprofessional competency in graduate education and training – implementation and evaluation of a case-based, interprofessional online seminar for pharmacists and physicians specializing in general practice

**DOI:** 10.3205/zma001842

**Published:** 2026-04-15

**Authors:** Sabine Gehrke-Beck, Nicole Zimmermann, Stefan Wind, Ulrike Sonntag

**Affiliations:** 1 Charité – Universitätsmedizin Berlin, corporate member of Freie Universität Berlin and Humboldt Universität zu Berlin, Institut für Allgemeinmedizin, Berlin, Germany; 2 Freie Universität Berlin, Abteilung Klinische Pharmazie & Biochemie, Berlin, Germany; 3 Apothekerkammer Berlin, Berlin, Germany

**Keywords:** education continuing, graduate education, interprofessional education, interprofessional relations, patient care team, online learning, medication review, medication therapy management

## Abstract

**Aim:**

Interprofessional competencies should be taught, over and above undergraduate education, in post-graduate continuing education. For better collaboration between the fields of medicine and pharmacy in regard to the safety of drug therapy in cases of polypharmacy, we designed an interprofessional training format for pharmacists and physicians specializing in general practice, which specifically aimed at improving the perception of roles and competencies by each professional group in order to improve everyday collaboration.

**Method:**

Based on an undergraduate teaching project in medicine and pharmacy, the Competence Center for Further Training in General Practice Berlin (Kompetenzzentrum Weiterbildung Allgemeinmedizin Berlin) and the Berlin Chamber of Pharmacists (Apothekerkammer Berlin) have developed a case-based, interprofessional seminar course for pharmacists (PH) independently pursuing continuing education and physicians pursuing specialization in general practice (GP trainees). Collaborative interdisciplinary discussions were held facilitated by interprofessional team teaching. The course, consisting of two teaching units, was offered in the evening in an easily accessible online format. The evaluation focused on participant satisfaction and changes in the perceptions of the other professional group.

**Results:**

A total of three online seminar sessions on different topics were held in 2023-24. Overall, 48 PH and 58 GP trainees participated. A total of 57 participants evaluated the course (response rate 54%). Of these, 55 would recommend the seminar course and all respondents assessed the interprofessional focus as helpful. They predominantly evaluated both the informational content and the opportunity for discussion as being appropriate. In the open-ended comments, becoming acquainted with the other professional group in the context of casework was identified as enriching. A total of 34 participants participated in a second evaluation after four weeks of professional work. Most of these respondents stated they were better able to discern the competencies of the other professional group; however, just barely half of them felt that interactions with the other professional group had improved. Individual participants, however, described difficulties making contact during routine work.

**Conclusion:**

An easily accessible, online seminar with interprofessional, case-based content received with a high level of acceptance and enabled interprofessional interaction. It led to an improvement in the perception of the other professional group's expertise, yet improvement in routine professional relations was limited.

## Zusammenfassung

**Zielsetzung:**

Interprofessionelle Kompetenzen sollten kontinuierlich auch über die Ausbildung hinaus in Fort- und Weiterbildung vermittelt werden. Zur besseren Kooperation zwischen Medizin und Pharmazie im Kontext der Arzneimitteltherapiesicherheit bei Multimedikation planten wir ein interprofessionelles Fortbildungsformat für Apotheker:innen(AP) und Ärzt:innen in Weiterbildung Allgemeinmedizin (ÄiW), welches insbesondere die Rollen- und Kompetenzwahrnehmung der jeweils anderen Berufsgruppe verbessern sollte, um die Zusammenarbeit im Berufsalltag zu verbessern.

**Methodik:**

Das Kompetenzzentrum Weiterbildung Allgemeinmedizin Berlin und die Apothekerkammer Berlin haben aufbauend auf einem Lehrprojekt im Medizin- und Pharmaziestudium ein fallbasiertes, interprofessionelles Seminar für sich fortbildende AP und ÄiW entwickelt. Im berufsgruppenübergreifenden Team-Teaching wurden kollaborativ fachübergreifend Falldiskussionen durchgeführt. Die Veranstaltung wurde niedrigschwellig online mit zwei Unterrichtseinheiten abends angeboten. Die Evaluation untersuchte die Zufriedenheit der Teilnehmenden (TN) und die Veränderung der Wahrnehmung der jeweils anderen Berufsgruppe.

**Ergebnisse:**

Es wurden 2023-2024 insgesamt drei online-Seminare mit unterschiedlichem thematischem Fokus durchgeführt. Insgesamt nahmen 48 AP und 58 ÄiW teil. 57 TN evaluierten das Seminar (Rücklauf 54%). 55 Evaluierende würden das Seminar weiterempfehlen und alle schätzten die interprofessionelle Ausrichtung als hilfreich ein. Sie beurteilten ganz überwiegend sowohl den Informationsgehalt als auch die Möglichkeiten zur Diskussion als angemessen. In Freitexten wurde besonders das Kennenlernen der anderen Berufsgruppe im Rahmen der Fallarbeit als bereichernd genannt. An einer erneuten Evaluation nach vier Wochen im Berufsalltag nahmen noch 34 TN teil. Ein Großteil gab an, die Kompetenzen der anderen Berufsgruppe besser beurteilen zu können, allerdings nur knapp die Hälfte, dass sich der Kontakt zur anderen Berufsgruppe verbessert hat. Einzelne TN beschrieben jedoch in Freitexten auch schwierige Kontaktaufnahmen im Alltag.

**Schlussfolgerung:**

Ein niedrigschwelliges online-Seminar mit interprofessioneller Fallbearbeitung trifft auf hohe Akzeptanz und ermöglicht einen berufsgruppenübergreifenden Austausch. Es führt zur verbesserten Wahrnehmung der Kompetenzen der jeweils anderen Berufsgruppe, verbessert aber den Kontakt im Berufsalltag nur begrenzt.

## 1. Introduction

### 1.1. Significance of interprofessional care and education

With the demographic shift, medical care will become more complex and interprofessional care increasingly more important [1], [2]. Interprofessional education is necessary to prepare students for interprofessional collaboration [2], [3], [4]. According to the WHO, “interprofessional education occurs when two or more professions learn about, from and with each other to enable effective collaboration and improve health outcomes” [5], [6].

Interprofessional competencies are anchored in the National Competency-based Catalogue of Learning Objectives in Undergraduate Medical Education and are being increasingly implemented at medical schools [7], [8], [9], [10], [11], [12], [13]. Nevertheless, many of the already implemented projects are only selectively anchored in the curriculum and accessible only to a portion of the students. Longitudinal integration has not yet been achieved in most undergraduate degree programs [4], [14]. Even after graduates have entered into professional practice, it does not make sense to offer continuing education and training in monoprofessional formats only; possibilities for interprofessional continuing education should also be created for this context [15], [16]. It is also the case that many of the people currently working in the health professions never experienced interprofessional education during their educations, and this should be remedied by addressing interprofessional collaboration for these target groups also.

### 1.2. General factors affecting interprofessional learning in graduate education

The implementation of interprofessional learning in graduate education and training is affected by different factors than those in undergraduate education. First, it is to be assumed that the relevance of daily collaboration between medical specialties is more directly apparent to practitioners than to students. Second, it is possible that behaviors and attitudes are more deeply entrenched and the degree of openness is lower toward less familiar training formats that, e.g., contain a higher proportion of interactive group work.

Like undergraduate education, graduate education and training are organized by profession, making the implementation of interdisciplinary courses more difficult [15]. Announcing and advertising such a course, as well as certification for professional credits, e.g., CME points for physicians and continuing education points for pharmacists (PH), must be taken into account for the different target groups.

Based on a successfully implemented, interprofessional education project for undergraduate medical and pharmacy students [17], through which an interprofessional network was established, we developed an adapted training format for the target groups of physicians and pharmacists.

### 1.3. Interprofessional cooperation when dealing with polypharmacy

Polypharmacy is associated with many negative outcomes; most evident is the correlation between additional hospitalizations and inappropriate prescriptions [18], [19]. The success of interventions that address polypharmacy and attempts to reduce negative consequences is varied [20]. In regard to interprofessional approaches, there are indications that polypharmacy can be reduced and complications avoided for the patients [21]. Not least, a study in Germany has shown that cooperative medication management could significantly decrease mortality [22]. General practitioners prescribe the majority of medications in the German healthcare system [23] making improved collaboration between PH and general practitioners especially relevant to improving patient care.

That said, collaboration between general practitioners and PH is scarcely established in routine practice in Germany. Studies show that, although each professional group has a rather positive perception of the other group and the advantages of collaborative work are understood, communication and interaction seldom take place [24], [25]. At the same time, a study has shown that older physicians have a more positive attitude toward and more contact with pharmacists than younger physicians do [25]. This was essentially explained as being that older medical practitioners have been able to cultivate more contacts and thus gather positive experiences.

### 1.4. Aim of the project

The aim of the course was to encourage collaboration and interaction between PH and GP trainees. The intention was to reach young physicians, in particular those who were just starting to gather experience with interprofessional collaboration in their routine work. The target group for the seminar course were PH and GP trainees. Young physicians were directly informed about the course via the Competence Center forFurther Training in General Practice Berlin.

Assuming that people basically have open attitudes, it seemed, above all, important to demonstrate in an interprofessional course the advantages that come with collaboration. The course learning objective was, specifically, to better appreciate the competencies of the other professional group and its role in providing patient care.

## 2. Project description

### 2.1. Teaching and learning methods

The course was developed according to the Kern cycle [26]. Becoming familiar with the competencies and role of the other professional group was defined as the main learning objective. To impart this, we planned for methodologically interactive and collaborative learning in small groups [27]. In an online setting, this can be achieved in an “online community of learning”, for example, in synchronous online, small groups and by requiring participants to actively contribute and then picking up on their contributions [28], [29], [30].

Small-group work was the most important pedagogical element of the seminar and enabled direct insight into the perspectives and expertise of the other professional group [27]. At the same time, the practical case was directly connected to providing patient care and clearly demonstrated the relevance of collaboration, thereby promoting acceptance of the educational format [31].

In addition, initial and supplementary insights into the knowledge and approaches of each professional group were conveyed in two brief input lectures focusing on the central theoretical and practical aspects of each professional group. The course was taught and moderated by instructors from both professions to increase the acceptance by both participating groups and to provide concrete role models engaged in cooperative and complementary collaboration.

When designing the schedule for the seminar course, time was allowed at the beginning to give all participants ample opportunity to speak and become acquainted with each other right away in the small groups. Furthermore, the introductory lectures were kept very short in the online format, slides only presented a minimum of text, and interactions via the chat function were encouraged to maintain attention spans.

The detailed schedule is presented in table 1 (Tab. 1).

Technical implementation was taken into account during the planning of the schedule. Tandem-teaching allows one person to take care of the technical aspects. Right before the seminar session began, all of the participants were told that the course would be interactive, that cameras and microphones are required and that participation must be via a PC or laptop.

### 2.2. Implementation

Contact with the Berlin Chamber of Pharmacists (Apothekerkammer Berlin) had already been established through an interprofessional education project in undergraduate studies of pharmacy and medicine [17]. The Competence Center for Further Training in General Practice Berlin (Kompetenzzentrum Weiterbildung Allgemeinmedizin Berlin), housed at the Berlin Charité's Institute for General Practice and Family Medicine (Institut für Allgemeinmedizin der Charité Berlin), organizes seminars for physicians to accompany postgraduate specialization in general practice. It was through these two organizations that the target groups were contacted and invited to participate, and the access details, handouts and evaluation were sent. Each organization covered half of the fees to pay the instructors. Despite the existing network and mutual willingness to jointly organize and conduct the seminar, the time needed for planning, with all of the necessary coordination from conception to the first seminar session, was about one year.

### 2.3. Evaluation concept

The evaluation covers several levels of Kirkpatrick’s evaluation model [32]. First, the participants’ satisfaction with the selected content and method was measured (Kirkpatrick level 1 “reaction” [33]), in particular, if the interprofessional format and the high percentage of interactive small-group work were perceived as meaningful. These questions were posed directly after the seminar by means of a self-developed questionnaire. Second, we wanted to survey and analyze the effects on participants' attitudes and routine professional work (Kirkpatrick levels 2 and 3, “learning” and “behavior” [34], [35]) to see if the perceptions of the other professional group and the interprofessional contact between them during the course of daily work had changed as a result of the seminar. To do this, a second evaluation was sent after four weeks. Both questionnaires contained questions with a six-point Likert scale and supplementary free-text fields.

## 3. Results

### 3.1. Participants

A total of three seminar sessions were held in 2023 and 2024 on the topics of depression, chronic pain, and antibiotic therapy. The example cases each entailed a patient with multiple pre-existing conditions and prescriptions so that, in addition to the main topic, other diseases and medications had to be discussed. All three of the sessions were quickly filled to capacity by both target groups; attempts were made to achieve a balanced number of participants from each profession. All of the sessions had a relatively high percentage (approximately 30% max.) of registered participants who did not attend in the end.

Overall, 48 PH and 58 GP trainees participated in the three sessions. Theoretically, it was possible that participants attended more than one seminar, since registering again was basically possible and we did not ask if the participants had already attended one of the previously held interprofessional seminars. During the last seminar, this question was asked as part of the greeting and only two of the participants stated that they had. All of the seminars were conducted by the same two instructors (NZ, SGB); at one of the sessions the instructors were assisted by a co-facilitator to handle the technical aspects.

### 3.2. Evaluation

**Figure 1:**
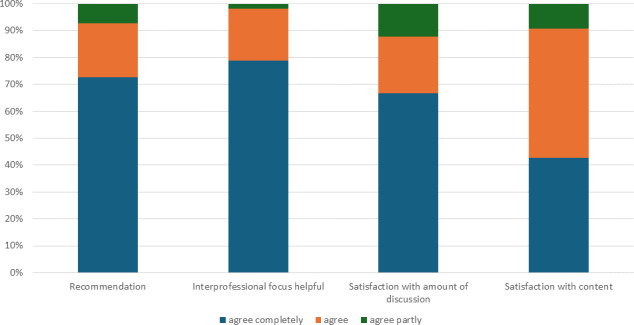
Post-seminar evaluation: Satisfaction with interprofessional orientation and methods

Of the 106 participants in total, 57 evaluated the seminar directly after the session ended (response rate 54%). A high percentage of those evaluating the seminar would recommend it to others, and all regarded the interprofessional focus as helpful. Most respondents also found the informational content to be appropriate and rated the opportunities for discussion as sufficient (see figure 1 (Fig. 1)).

Interacting with the other professional group and cooperating on the case were rated especially positively in the open-ended comments (n=11) (see table 2 (Tab. 2)). Individual participants wished they had received more information and more input (n=3); others would have liked to have had even more time for discussion (n=4). Several critically mentioned technical issues regarding implementation (n=3).

**Figure 2:**
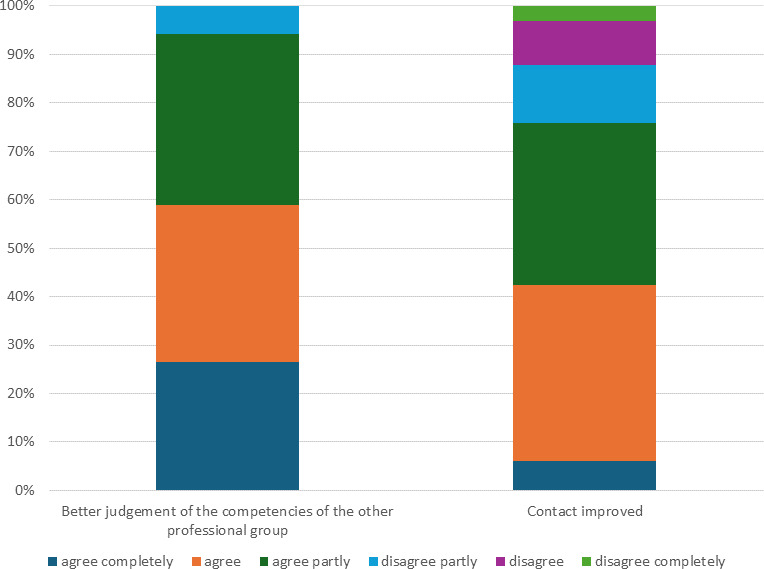
Evaluation after 4 weeks: Perception of other professional groups and contact in everyday working life

A total of 34 participants took part in a second evaluation after four weeks of routine work (response rate 32%). The majority stated they were better able to judge the competencies of the other professional group; however, less than half agreed that the relations with the other professional group had clearly improved (see figure 2 (Fig. 2)).

Not only were the interactions with the other professional group described (n=3) in the free-text fields, but also the other effects on daily work as a result of the course (n=2) (see also table 3 (Tab. 3)).

## 4. Discussion

### 4.1. Summary

The evaluation results show a high level of satisfaction on the part of the participants with the training format and particularly with the methodological approach, the focus on collaborative and active learning techniques and interprofessional orientation. Whereas most of the interaction with the other professional group was perceived as positive and enriching, some participants expressed the desire for more information and presentations. Some participants experienced technical difficulties.

The participants stated they had a better perception of the other professional group's competencies in their everyday work; however, the effects on developing relations vary.

### 4.2. Comparison with the literature

The improved perception of the other professional group's expertise shows that collaborative case analysis is a method which, even at a low intensity and in a single session, can lead to changes. This confirms not only general assumptions about the effectiveness of active and collaborative learning [36], but also methodological recommendations [4], [27] and studies on interprofessional education [31]. Although the recommendations and outcomes are geared toward undergraduate medical education, they can presumably be transferred to graduate education and training. In addition to activating and cooperative methods, centering the patient and case analysis also help to create effective learning situations. This has been observed in both undergraduate and graduate education [31], [37].

Technical difficulties were experienced by individual participants, and the online moderation of the case analyses was technically and methodologically challenging. This has also been described for comparable projects. Implementing interprofessional sessions in an online format is nevertheless considered practically feasible and valuable, even if they need special facilitation and an adapted methodology [29], [30], [38]. For this reason, successful moderation of an interprofessional online course requires particularly thorough preparation and methodological expertise [30], [38], [39], which is consistent with our impression during implementation. Precisely in the case of interprofessional education, an online course provides an easily accessible way for all of the professional groups to participate [28], [40], [41], [42]. One important aspect of planning for several professional groups is selecting course locations and times that ensure all participants feel equally comfortable [27]. It has been found that the online setting can make interpersonal encounters easier because professional divides and hierarchies are perceived less strongly [28]. An online course is thus the most likely space that is equally familiar to all participants and does not represent any particular profession's usual educational setting. That said, it is possible that differing prerequisites arise here; for instance, the use of video software that is well known by one professional group and less so by another. That interprofessional learning is also effective in the online setting has been demonstrated for different professions and in different formats [40], [41], [43], [44], [45]. Due to the very real advantages, it seems sensible to address the challenges of online implementation and in doing so create the option of having easily accessible, low-barrier interprofessional learning opportunities [41], [42], [46].

### 4.3. Limitations

It must be noted as a limitation that presumably the people voluntarily participating in the seminars were those who are more open to interprofessional collaboration, and it cannot be ruled out that individual participants also attended multiple sessions. Only a little more than half of the participants evaluated the course immediately after the seminar session, and only about one third after four weeks. In particular, the statements regarding the effects of the course on daily work are therefore uncertain. Where changes did occur in the routine work setting, it is difficult to define in what form the course was effective on its own since many other factors can influence behavior. Comparable studies would be desirable for closer investigation of a behavior change. The investigation of the learning effect is based only on the participants' subjective statements and the study did not have an objective pretest-posttest design.

### 4.4. Project goals attained und persisting issues

With the case-based interprofessional course, we were able to establish a format which was easily accessible, very well sought after and accepted. All of the scheduled seminars reached full registration capacity for both professional groups. Although case-based courses with a small amount of time dedicated to imparting information was not the familiar educational format for the participants, their level of satisfaction with the selected methodological approach was high and the interprofessional casework was experienced very positively by a predominant majority. Individual participants were dissatisfied with or surprised by the small amount of time spent lecturing or imparting information. Even though clear information was given about the course’s interactive format when it was first announced, it is possible that this must be more clearly stated to communicate that it is not a conventional lecture course and active participation is intended. Also, continued improvement to the technical procedures appears wise. During the course, we already included an additional person on the team to oversee the technical facilitation. Additional possibilities, such as starting the technical check-in earlier, not allowing latecomers to join, or switching to another webinar platform could be tried.

Whether improvement in the relations between the professional groups in routine practice can come about is unclear. Individual open-ended comments indicate that contextual factors can stand in the way of change. It is quite conceivable that a participant who has changed their perception or attitude as a result of the course has only a limited scope of action in a professional environment that remains otherwise unchanged. Changes in everyday professional behavior could be specifically talked about and strategies for overcoming the existing barriers could be collaboratively identified and discussed as part of the seminar. The transfer could also happen more directly when training programs bring local collaborators into contact with each other, e.g., in a local interprofessional quality circle. In this case, classroom-based courses with fewer obstacles could be feasible. Widespread implementation of such formats, however, would take effort and come up against, in part, profession-specific prejudices. The effectiveness of a training program by itself will therefore always be limited as long as the structures shaping professional practice go unchanged.

## 5. Conclusions and project continuation

In summary, the methodological approach using case-based, interactive interprofessional learning in the context of graduate education is viable and evaluated positively by the participants. A change in the perception of the other professional group's expertise can be achieved after a single seminar session. It is harder to influence the effects on the relations between the professional groups and an improved collaboration in routine practice because these take place in otherwise unchanged structures. Other continuing educational formats that bring professionals already active in providing patient care into contact with each other and that create shared learning opportunities can impact more directly here, but they also require more effort to implement and reach smaller target groups. For this reason, it seems sensible to act on various levels. The format developed and presented here should therefore be continued and supplemented with additional offerings.

## Acknowledgements

Thanks to Alexandra Blehe (Apothekerkammer Berlin) and Daniela Nickel and Kahina Toutaoui (Kompetenzzentrum Weiterbildung Allgemeinmedizin Berlin) for organizing the seminars, which in general entailed much greater effort than similar monoprofessional courses, and without whose willingness to make it happen, this project would not have been possible.

## Authors’ ORCIDs

- Sabine Gehrke-Beck: [0000-0002-6221-2813]
- Nicole Zimmermann: [0009-0002-3157-814X]
- Ulrike Sonntag: [0000-0001-9576-2734]

## Competing interests

The authors declare that they have no competing interests. 

## Figures and Tables

**Table 1:**
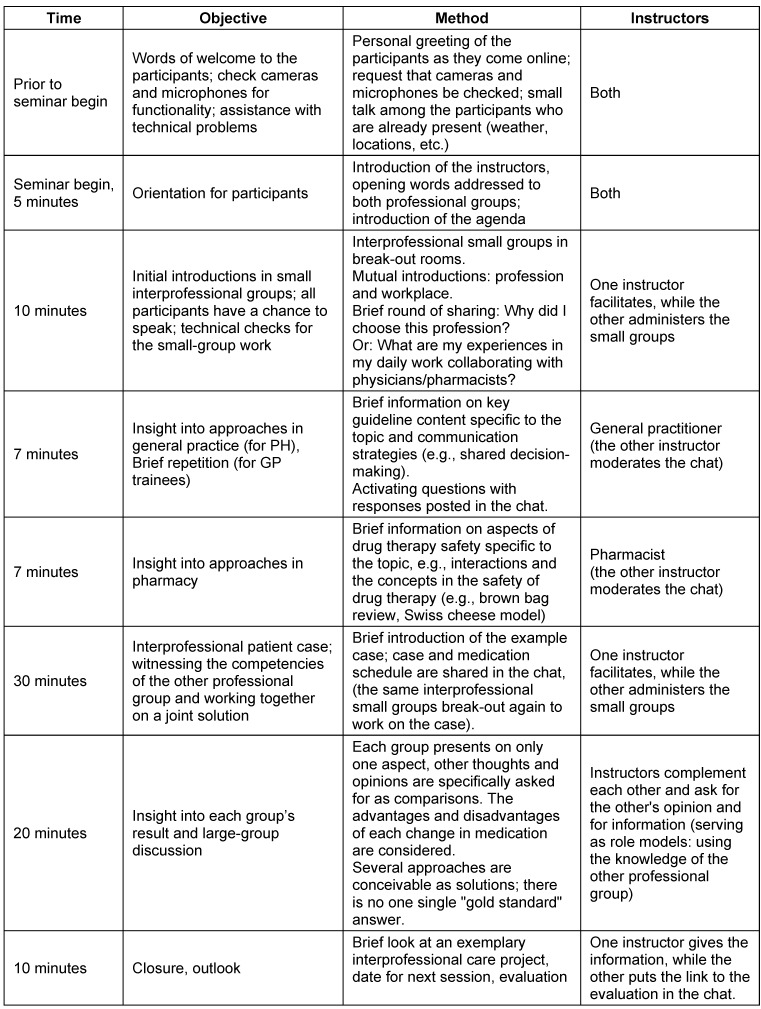
Detailed schedule

**Table 2:**
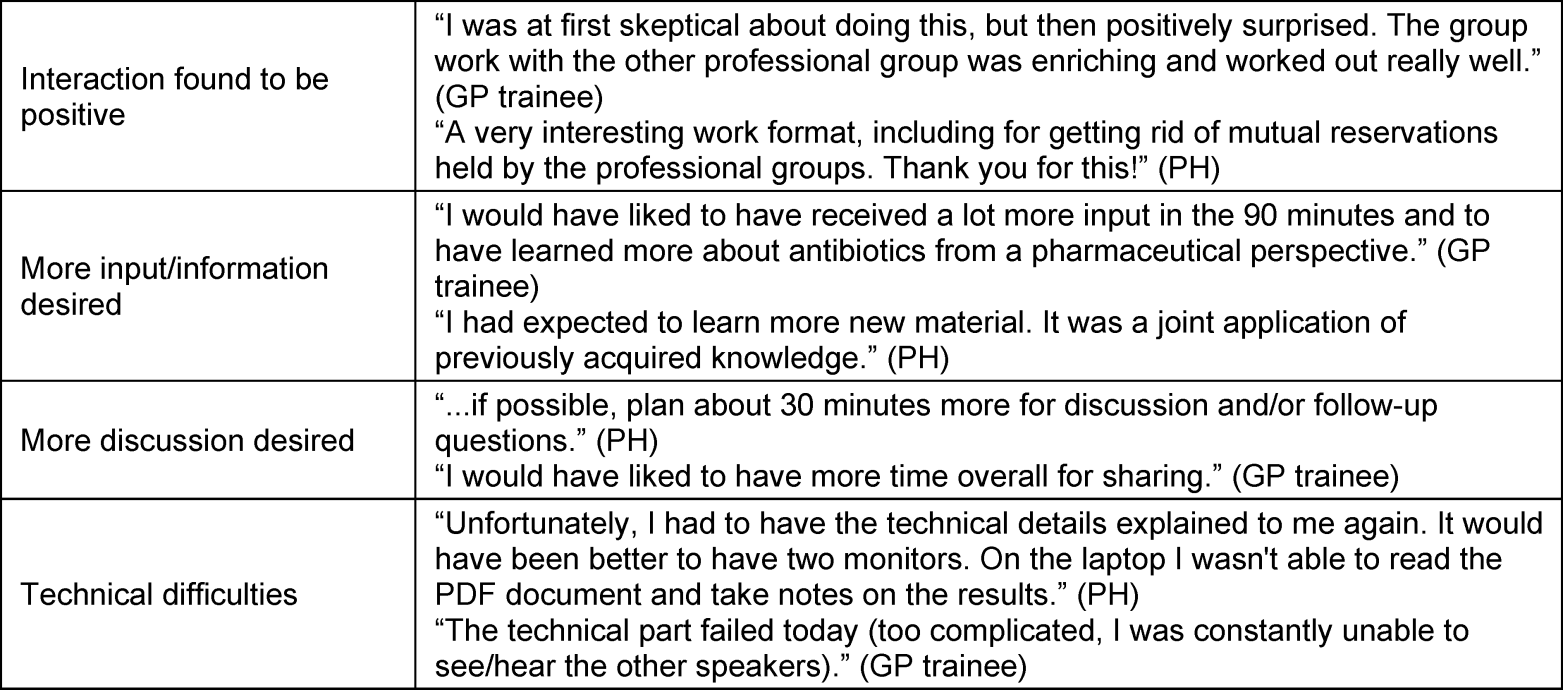
Examples from the open responses

**Table 3:**
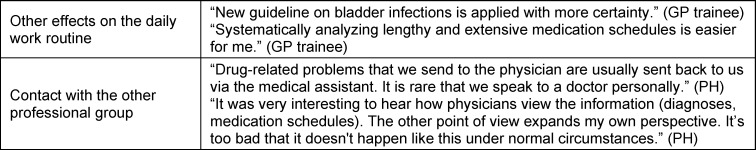
Open responses concerning relations with the other professional group
